# Dunking rusk: innovative food soaking behaviour in Goffin's cockatoos (*Cacatua goffiniana*)

**DOI:** 10.1098/rsbl.2023.0411

**Published:** 2023-12-13

**Authors:** J. S. Zewald, A. M. I. Auersperg

**Affiliations:** Comparative Cognition, Messerli Research Institute, University of Veterinary Medicine Vienna, Medical University of Vienna and University of Vienna, Vienna, Austria

**Keywords:** innovation, dunking, foraging, food preparation

## Abstract

Foraging innovations in animals involving the processing of resources that are already edible in an unprocessed state, yet of improved quality in a processed state, are rare but important to study the evolution of food preparation. Here, we present the first scientific report of food dunking behaviours in parrots by Goffin's cockatoos, a model species for innovative problem solving. Observations during lunch showed seven out of 18 cockatoos placing their food into water and soaking it prior to consumption. This was largely done with dry rusk which was eaten almost exclusively when dunked. Furthermore, their transport effort and waiting times before retrieving food from the water indicate their willingness to invest considerable time to prepare a soaked rusk piece of a higher texture quality. Our present results suggest that the function of this behaviour is to soak the food. Because only some individuals dunked food and dunking has not been observed in the wild, we believe this to be a spontaneous foraging innovation either by one or multiple individuals.

## 1. Introduction

Foraging innovations, defined as ‘the use of a new food type or novel foraging technique' [1] correlate with several (relative) brain size measurements [1–3] and cognitive skills [4,5]. Among foraging innovations, food technique innovations in which a species uses a novel method to gain or alter a food item [6] are particularly suitable predictors for residual brain size [7]. Among them, scientific reports on innovative food preparations (i.e. improving the state of a resource that is edible in its present state) are rare and most reports are supported by limited footage or purely anecdotal [8]. This makes it difficult to confidently infer the distribution of the behaviours in the population and their function. However, detailed descriptions of such behaviours could provide significant stepping stones towards understanding the evolution of food preparation.

A prime example of such food preparation is dunking behaviour, dipping of food in liquid before ingestion [9]. Five main reasons for food dunking behaviour have been suggested: first, food dunking can soak and/or soften hard, dry foods [9,10] and thereby improve the texture quality of the food or help with ingestion. Second, food dunking could clean the food [11,12] by washing off dirt, coatings and toxins on the food before ingestion. Third, dunking behaviour in flavoured liquids, like salt water, could improve the taste quality and season the food [13,14]. Fourth, dunking live prey could be a method to drown them [15,16]. Fifth, food dunking could be a way to transport liquid away, like a sponge. For example, to bring liquid to individuals that cannot access it themselves [17]. However, explanations for almost any food dunking behaviour can be diverse and for making clear inferences on the individual's goals, experimental data are almost indispensable [9,11,12,18,19].

In this study, we present a series of systematic observations following up on the discovery of food dunking behaviour in a group of captive Goffin's cockatoos (*Cacatua goffiniana*), a model species for technical innovations and flexible problem-solving [20–22]. After observing food dunking behaviour in three of our birds (Kiwi, Pipin, Muki) during lunch feedings, we decided to target the following questions: which food items are actively transported and dunked by the birds? How widespread is this behaviour in the group? How long do the birds leave the food in the water before consuming it? Soaking has been suggested to be the main goal of food dunking in non-predatory birds [23]. If this would also be the goal of the cockatoos, we would expect that they would actively transport mainly dry food to the water sources and wait for their food to soak before eating it. Its innovativeness could be reflected by it being limited to specific individuals and not be shown population wide.

## 2. Methods

### (a) Subjects and housing

A group of 18 Goffin's cockatoos (nine males, nine females; table 1) are permanently housed in an enriched aviary (indoor: 45 m^2^, 3–6 m high; outdoor: *ca* 200 m^2^; 3–4.5 m high) at the Goffin Lab in Lower Austria. Because of some social incompatibility, five enriched cages (1 × 1×2 m; Montana Cages) were available in the indoor aviary. From 08.00 to 14.00 Figaro was kept in a cage and from 14.00 to 08.00 Kiwi, Pipin, Moneypenny, Zozo and Olympia were in a cage each (the rest of the birds always remained in the main area). This way all birds could to fly, play and interact with the rest of the group for several hours.

**Table 1.** Subject details. The subject's name, age, cage situation, number of food dunks and the median time a subject left the food in the water before eating together with the median absolute deviation (MAD).

individual	sex	age (y)	in cage?	total number of dunking events	time in water for rusk (s)	
					median	± MAD
Pippin (focal)	♂	14	during lunch	35	31.2	18.5
Kiwi (focal)	♂	12	during lunch	53	13.2	8.9
Moneypenny	♀	12	during lunch	5	6.0	1.5
Olympia	♀	12	during lunch	0	–	–
Zozo	♂	12	during lunch	0	–	–
Figaro	♂	15	in morning	0	–	–
Muki	♂	11	no	34	4.8	2.6
Jane	♀	5	no	3	34.5	43.4
Dory	♀	1	no	1	4.0	–
Rosy	♀	1	no	8	6.1	2.9
Fini	♀	15	no	0	–	–
Heidi	♀	12	no	0	–	–
Muppet	♂	12	no	0	–	–
Doolittle	♂	11	no	0	–	–
Mayday	♀	11	no	0	–	–
Irene	♀	5	no	0	–	–
Titus	♂	5	no	0	–	–
Renki	♂	2	no	0	–	–

In the aviary, two cylindric tubs were present (Ø75 cm, h: 20 cm), for drinking, bathing and potentially food dunking. Around 10.00, the birds received breakfast (egg, noodles, potatoes or cauliflower with fruit and soy yoghurt) and lunch was given around 14.00 after we switched the birds in the cages. Lunch consisted of rusk (twice-baked bread/toast; Feldbacher Zwieback Klassik), dried banana chips, dried coconut chips, cornflakes, thistle seeds, fennel seeds, a dried berry mix, dried apple pieces and bird pellets (Versele-Laga Nutribird P15 Original), often mixed with mineral supplements. In the aviary, lunch was provided in four ceramic bowls (Ø30 cm) with an approximate distance of 1 m to the water tubs. In the cages, water and lunch were provided in two aluminium bowls (Ø15 cm) approximately 20 cm from each other.

### (b) Measurements

#### (i) Observations

During 12 days in July and August of 2022, we observed the first 15 min after lunch was served. During these observations, we focal-sampled Kiwi and Pipin because they were socially undisturbed in their cages, while also sampling dunking behaviour of the other birds during that time. The other caged birds were not focal-sampled because they were irregularly in their cages. Sessions were video recorded with a smartphone (Samsung Galaxy A52 5G), and later analysed with the observation program BORIS [24]. We coded who and what food item they dunked, whether they ate the dunked food and how long it was left in the water (for details, see electronic supplementary material, S1). Sometimes, the birds only partially took the food out or placed it back in the water after eating some of it. Here, we report the minimal amount of time the birds left the food in the water before eating it. For the focal individuals, we noted whether they also ate the soaked food items dry.

#### (ii) Soaking measurements

We dunked 20 pieces of rusk of similar sizes presented to the birds (average piece size *ca* 7 cm^2^, average weight = 1.47 ± 0.34 g) in water and every 5 s we took three-time measurements: (1) when the bottom of the piece was wet and soft (i.e. no longer crushable), (2) when the core of the piece was soft and (3) when the piece fell apart (with a maximum time of 180 s). We tied a small string around the pieces, so we could reliably dunk and retrieve them. Additionally, we measured the weight increase for 20 pieces each 5 s, to quantify the water absorption by the rusk. We considered the rusk to be saturated when their water weight gain stopped changing more than 1 g.

#### (iii) Statistical tests

To analyse whether the cockatoos dunk one food type more than others, we used a generalized linear mixed model with a negative binomial distribution. We used the number of dunked food items as response, food type as predictor and subject as a random intercept (see S2). To determine individual preferences to eat the food dunked or dry, we used binomial tests with a Holm–Bonferroni *p*-adjustment [25] to correct for multiple testing.

## 3. Results

### (i) Distribution of dunking

**Figure 1.**
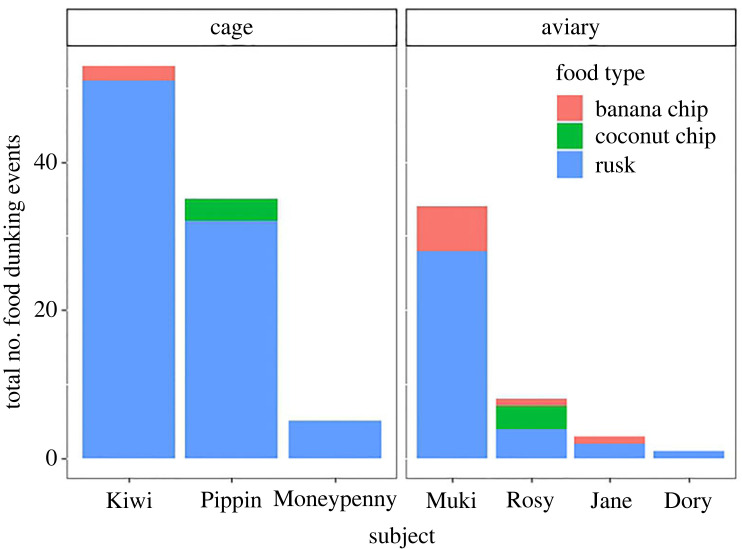
Total frequency of dunking behaviour in group. The subjects that dunked are on the *x*-axis and the colours represent the dunked food items.

**Figure 2.**
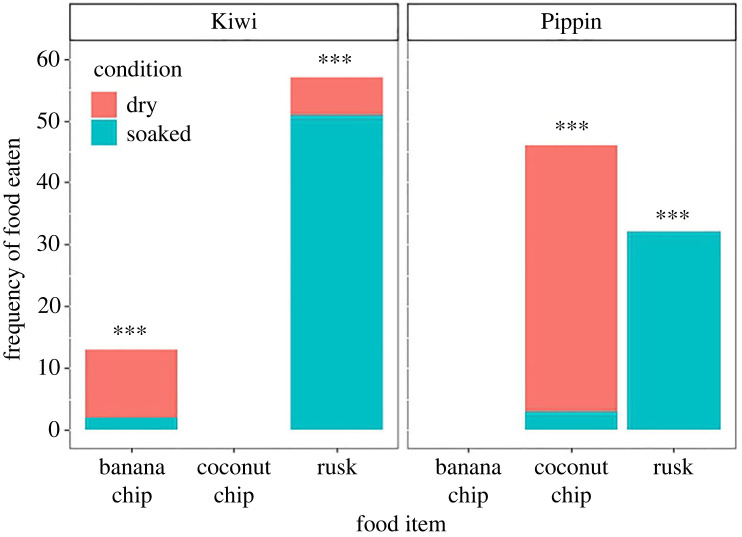
Individual preferences for eating food dry or soaked. The food items that had been dunked are on the *x*-axis and the colours represent whether the birds ate this food item dry or soaked.

We observed seven out of 18 cockatoos dunking food in the water (figure 1; electronic supplementary material, video S3). They dunked three food types: rusk (*n* = 123), dried banana chips (*n* = 10) and dried coconut chips (*n* = 6). Other food types, like apple pieces, dried berries, seeds or pellets, were never dunked during our observations. There was a trending effect of type of food item on dunking behaviour (figure 1; neg.binom. GLMM, X^2^ = 5.3756, *p* = 0.068) with them having a significant preference to dunk rusk over the coconut and banana chips (Tukey HSD *post hoc*, *p* < 0.001, *p* < 0.001 respectively), but not between the coconut and banana chips (*p* = 0.582). For the focals (Kiwi and Pipin), we were able to compare how often they ate the dunked foods dry or soaked (figure 2). For the rusk, both individuals had a significant preference to eat the rusk soaked (Pipin: 32/32, binomial test, *p* < 0.001; Kiwi: 51/57, binomial test, *p* < 0.001), but this preference was reversed for the coconut and banana chips (coconut Pipin: 2/11, binomial test, *p* = 0.025; banana Kiwi: 3/43, binomial test, *p* < 0.001).

### (ii) Effort

If the birds' goal was to soak the rusk, they would need to wait for water to be absorbed by it. Our own soaking measurements showed that rusk pieces got a soft bottom after an average of 19.5 ± 3.2 s, a soft core after 30.8 ± 4.7 s and started to fall apart around 82.8 ± 50.1 s. Furthermore, the pieces were saturated after 65.8 ± 12.8 s, having absorbed 9.7 ± 2.5 ml of water (electronic supplementary material, figure S1). Before eating it, the cockatoos left their rusk in the water for 22.89 ±25.48 s (table 1 for individual values), although the average time for the caged birds (26.44 ± 24.02 s) seemed longer than the ones in the aviary (13.35 ± 26.75 s) (see also electronic supplementary material, S4).

Additionally, the focals sometimes invested physical effort by climbing to transport food that had fallen to the bottom of the cage to their perch (1.2 m higher) where the water bowls are located (electronic supplementary material, video S5). Most of the food they climbed with was rusk (*n* = 20) and sometimes coconut (*n* = 3) or banana chips (*n* = 1). However, the coconut and banana chips were always eaten dry when the birds reached the perch. Most of the rusk was dunked before eating (*n* = 16). In the other cases, the food was not eaten (*n* = 2) or it had been lying in a puddle at the bottom of the cage before the birds climbed up (*n* = 2).

## 4. Discussion

We found that seven out of 18 Goffin's cockatoos in our group were actively transporting food to water sources and dunking it. The behaviour seemed to be mainly targeted at rusk, a dry and hard food type that easily absorbs water and adopts a soggy texture. Our focal birds ate rusk almost exclusively after dunking it.

Going back to the five previously proposed goals of dunking behaviour in animals, we believe that drowning prey and water transport both seem unlikely as all subjects have ad libitum access to water sources nearby (particularly in the cages where they sat near the water; electronic supplementary material, video S3) and no live prey are involved. Seasoning behaviour also seems implausible as they dunk food in fresh, unflavoured tap water. Washing the food to get rid of the supplementary minerals also seems unlikely as we would expect the animals to dunk all food items equally, and not mostly rusk, and because we also observed dunking on days without mineral supplement. Because the birds eat rusk nearly always dunked, soaking seems to be the likely function for the behaviour, confirming previous observations in other bird species [9,10,23].

Initially, we observed three individuals dunking and had no data on other individuals before this study. Therefore, the initial onset and whether these were individual innovations or (partially) socially transmitted remains unknown. However, dunking was limited to seven out of 18 individuals and is thus not present at a species or even at a population-wide level. So far, food dunking has not been observed in the wild Goffin populations, potentially due to a lack of open water sources or soakable food (B. Mioduszewska; T. Rößler, personal communication 2022; 2023). Therefore, this likely seems to be a foraging innovation. Alternatively, we cannot fully exclude the possibility of seven individuals preferring soaked food while the rest of the population prefers dry, which could also explain the limited number of individuals dunking.

Previously, it has been suggested that innovating dunking behaviour is relatively simple in captivity where the circumstances are favourable (food availability, open water source nearby, minor risk/cost of kleptoparasitism) [10,12], which could also explain why relatively more caged birds dunk (60%) than in the aviary (31%). Nevertheless, dunking does require a level of impulse control and temporal discounting to soak the rusk and inhibit immediate consumption [19]. The cockatoos invested transport effort and an average time of 23 s to let the rusk soak, which was long enough to soften the bottom of the rusk. In a previous delay of gratification task, the cockatoos waited on average 29.61 ± 28.21 s for an increase in food reward quality [26]. This is comparable to the time they waited here for a reward with a softer texture quality. This could also explain why dunking of the coconut and banana pieces is not preferred as these would take more time to rehydrate. Although the average waiting time seemed to be shorter for the birds in the aviary, potentially because of kleptoparasitism [10] (electronic supplementary material, video S6), we were unable to statistically investigate this due to the limited number of dunking birds. However, climbing with rusk by our focals to the water also shows the effort they are willing to invest to gain soaked rusk: the beak is normally used to stabilize the body during climbing but is somewhat constrained when holding the rusk. Thus, transporting food during a 1.2 m (perch height) climb is likely to be ergonomically strenuous.

In conclusion, this is the first scientific report of dunking behaviour in a parrot species. Although the behaviour itself is likely more common among (pet) parrots (reflected by internet videos and reports by owners), systematic reports are necessary as owner reports are prone to misinterpretations, human influencing and lacking controls. Our observations suggest a foraging innovation of selective dunking by captive Goffin's cockatoos with the goal of soaking food. This dunking behaviour requires several components of primordial forms of planning such as impulse control and temporal discounting to actively produce a qualitative benefit. It also once more reflects the innovativeness of this parrot species, now also in a food preparation context. Future research should focus on the change and diffusion of this behaviour in the group [13].

## Data Availability

Our data, R code and R workspace are provided in the electronic supplementary material [27].
